# Unraveling the efficacy of verbascoside in thwarting MRSA pathogenicity by targeting sortase A

**DOI:** 10.1007/s00253-024-13202-6

**Published:** 2024-06-05

**Authors:** Xingchen Li, Yingying Hou, Haoyan Zou, Yueying Wang, Yueshan Xu, Li Wang, Bingmei Wang, Ming Yan, Xiangyang Leng

**Affiliations:** 1 Changchun University of Chinese Medicine, Changchun, China; 2 The First Affiliated Hospital of Zhengzhou University, Zhengzhou, China

**Keywords:** Methicillin-resistant *Staphylococcus aureus*, Sortase A, Antivirulence, Verbascoside

## Abstract

**Abstract:**

In the fight against hospital-acquired infections, the challenge posed by methicillin-resistant *Staphylococcus aureus* (MRSA) necessitates the development of novel treatment methods. This study focused on undermining the virulence of *S. aureus*, especially by targeting surface proteins crucial for bacterial adherence and evasion of the immune system. A primary aspect of our approach involves inhibiting sortase A (SrtA), a vital enzyme for attaching microbial surface components recognizing adhesive matrix molecules (MSCRAMMs) to the bacterial cell wall, thereby reducing the pathogenicity of *S. aureus*. Verbascoside, a phenylethanoid glycoside, was found to be an effective SrtA inhibitor in our research. Advanced fluorescence quenching and molecular docking studies revealed a specific interaction between verbascoside and SrtA, pinpointing the critical active sites involved in this interaction. This molecular interaction significantly impedes the SrtA-mediated attachment of MSCRAMMs, resulting in a substantial reduction in bacterial adhesion, invasion, and biofilm formation. The effectiveness of verbascoside has also been demonstrated in vivo, as shown by its considerable protective effects on pneumonia and *Galleria mellonella* (wax moth) infection models. These findings underscore the potential of verbascoside as a promising component in new antivirulence therapies for *S. aureus* infections. By targeting crucial virulence factors such as SrtA, agents such as verbascoside constitute a strategic and potent approach for tackling antibiotic resistance worldwide.

**Key points:**

*• Verbascoside inhibits SrtA, reducing S. aureus adhesion and biofilm formation.*

*• In vivo studies demonstrated the efficacy of verbascoside against S. aureus infections.*

*• Targeting virulence factors such as SrtA offers new avenues against antibiotic resistance.*

**Graphical abstract:**

**Figure:**
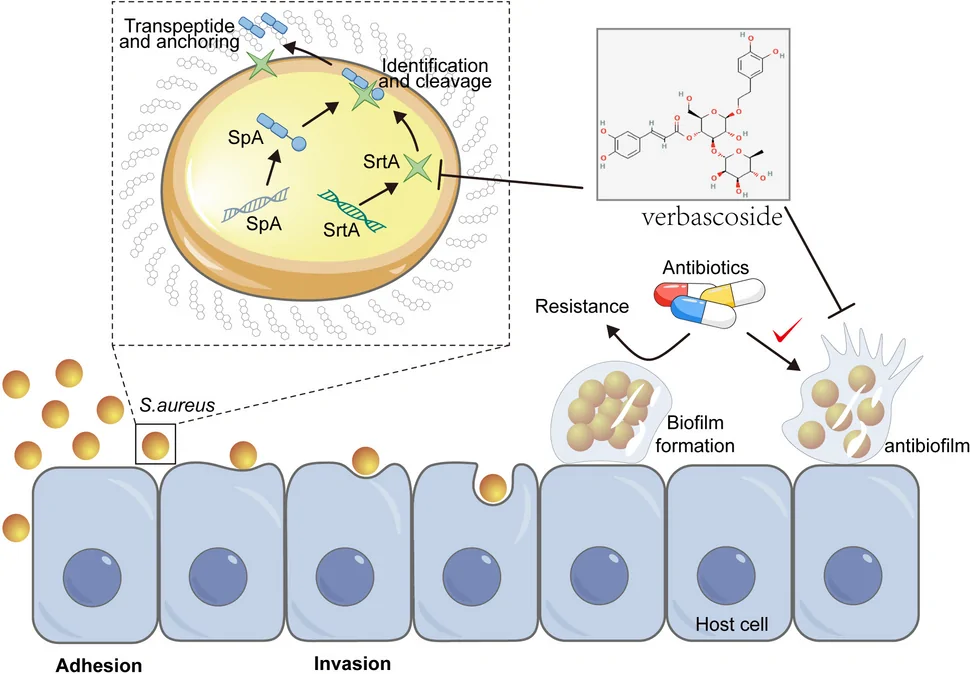

**Supplementary Information:**

The online version contains supplementary material available at 10.1007/s00253-024-13202-6.

## Introduction

In the realm of nosocomial infections, gram-positive bacteria, notably methicillin-resistant *Staphylococcus aureus* (MRSA), pose a significant challenge to clinical practitioners (Shulga and Kudryavtsev 2022). *S. aureus*, a gram-positive bacterium, plays a pivotal role in a myriad of human infections, ranging from superficial skin infections to invasive deep tissue infections (Bai et al. 2022). Initially, conventional antibiotics and antimicrobial agents were considered promising for curtailing *S. aureus* infections. However, the imprudent and irrational use of antibiotics has facilitated the emergence and dissemination of resistant strains under conducive conditions. Consequently, the emergence of resistant strains such as MRSA and vancomycin-resistant *S. aureus* (VRSA) often renders antibiotic therapy ineffective (Lázár et al. 2022). Despite a decrease in MRSA infection rates, the ramifications of such infections remain severe. This underscores the urgent need for novel therapeutic strategies that mitigate drug resistance while effectively reducing bacterial infections. The virulence factors secreted by *S. aureus*, including surface proteins and toxins that promote bacterial adhesion, tissue invasion, destruction, and evasion of host defenses, are intimately linked to its pathogenicity (Nandhini et al. 2022). The proposed antivirulence strategy offers a fresh perspective for slowing the development of bacterial resistance to conventional antibiotics. Unlike traditional antibiotic therapies, antivirulence strategies do not exert survival pressure on *S. aureus* (Peng et al. 2022). By targeting the bacterium’s virulence factors, these antivirulence agents weaken its pathogenicity by disarming rather than annihilating it (Willis et al. 2022).

Bacterial adhesion to host tissues, a critical initial step in infection and colonization, is essential for the formation of biofilms that protect pathogens from host immune attacks. Inhibiting the bacterial adhesion process is considered a promising antivirulence approach. In gram-positive bacteria, pili, covalently linked to the peptidoglycan layer, are structural protein motifs. These cell wall anchoring (CWA) proteins play a principal role in adhesion, as they facilitate the localization of host extracellular matrix proteins, such as collagen, fibrinogen, and fibronectin (Berry et al. 2022). A subfamily of CWAs, microbial surface components recognizing adhesive matrix molecules (MSCRAMMs), are covalently linked to peptidoglycan via a unique sortase transpeptidase known as sortase A (SrtA). Sortase, a ubiquitous enzyme in gram-positive bacteria, is pivotal to bacterial virulence (Morales-Laverde et al. 2022). SrtA, a prototypical sortase, has garnered increased amounts of attention for its potential as an antivirulence therapeutic target. It is a membrane-bound transpeptidase that comprises an N-terminal transmembrane region and a C-terminal catalytic domain. SrtA recognizes MSCRAMMs through its sorting signal, which is composed of the LPXTG motif, where X represents any amino acid. The anchoring process begins with the recognition of the LPXTG motif, followed by a trans-esterification reaction that cleaves the peptide bond between the threonine and glycine residues of the sorting motif, forming a thioester acyl-enzyme intermediate (Cascioferro et al. 2015). A second transpeptidation reaction mediated by SrtA then occurs between the thioester intermediate and the pentaglycine (Gly5) unit of lipid II on the cell wall, covalently anchoring the product to the cell wall, where it facilitates bacterial adhesion to host cells and tissues (Hernández-Cuellar et al. 2023).

The inhibition of SrtA has been demonstrated to result in reduced biofilm formation in some *S. aureus* strains and a loss of binding activity to fibronectin, fibrinogen, and immunoglobulin G, thereby diminishing bacterial virulence (Hansenová Maňásková et al. 2022). Furthermore, several characteristics of SrtA make it an outstanding target for preventing bacterial virulence. Primarily, SrtA is not essential for bacterial survival or growth, a fundamental characteristic of an antivirulence target. Importantly, there are no human homologs of SrtA, suggesting the possibility of selective inhibition (Audah et al. 2022). Finally, as a membrane-associated protein, SrtA is a relatively accessible target, as inhibitors do not need to traverse the bacterial outer membrane to enter the cytoplasm. To date, several SrtA inhibitors have been identified that are derived from various natural products, small organic molecules, and peptides (Tian et al. 2022; Wang et al. 2022; Tian et al. 2023; Wang et al. 2023). With their diverse, novel structures, rich biological activities, and minimal side effects, natural compounds have always been a primary choice in new drug development (Zhao et al. 2022).

Verbascoside, a phenylethanoid glycoside initially isolated from Forsythia but also found in several other plant species, is produced through in vitro plant culture systems, including genetically transformed roots (so-called “hairy roots”). Verbascoside is hydrophilic in nature and has pharmacological activities that are beneficial to human health, including antioxidant, anti-inflammatory, and antitumor effects. Additionally, it promotes extensive wound healing and has neuroprotective effects. However, there have been no reports on the antivirulence effects of verbascoside. Subsequently, we evaluated the antimicrobial activity of *S. aureus* SrtA and the associated influence of verbascoside on virulence in vitro, demonstrating its potential as an inhibitory compound against SrtA.

Our investigation revealed that verbascoside significantly impaired the SrtA-mediated anchoring of MSCRAMMs to the cell wall, thereby inhibiting the adhesion of *S. aureus* to host tissues. This interference with the bacterial adhesion process is pivotal for preventing the formation of biofilms and reducing the ability of bacteria to evade the host immune response. These findings suggest that verbascoside, by targeting SrtA, impedes the virulence of *S. aureus*, suggesting that this novel therapeutic approach diverges from conventional antibiotic strategies. This aligns with the growing interest in developing antivirulence therapies to address the burgeoning issue of antibiotic resistance.

## Materials and methods

### Reagents and materials

The *S. aureus* USA300 (ATCC BAA-1717) and *S. aureus* Newman (ATCC 25923) strains were obtained from the American Type Culture Collection (ATCC) in Manassas, Virginia, USA. *Escherichia coli* DH5α and BL21 (DE3) strains were purchased from Tiangen Biotech (Beijing) Co., Ltd. Clinical isolates SA1B3B of MRSA and SA37 of clinical isolate MSSA were preserved in the laboratory. In our laboratory, we established cultures of the *S. aureus* USA300 strain lacking the SrtA gene *(ΔsrtA)* and harboring the pET28a-*srtA* plasmid. *Escherichia coli* and *S. aureus* were cultivated in Luria–Bertani (LB) and tryptone soy broth (TSB), respectively, at 37 °C. The chemical agent dimethyl sulfoxide (DMSO) was obtained from Biotechnology (Shanghai, China). Verbascoside was sourced from Letian Mei Biology Co., Ltd., based in Chengdu, China. Additionally, the specialized fluorescent substrate peptide Abz-LPATG-Dap (Dnp)-NH_2_ was custom synthesized by LifeTein, LLC, which operates outside of Beijing, China.

### SrtA mutant plasmid construction and protein purification

Both the original and mutant versions of the pET28a::*srtA* plasmid were introduced into the *Escherichia coli* BL21 (DE3) strain. This was achieved through the heat shock transformation technique using chemically competent cells supplied by Tsingke Biological Technology. The bacterial cultures were allowed to grow until an optical density (OD_600_) of 0.8 was reached. Subsequently, isopropyl β-D-1-thiogalactopyranoside (IPTG) was added to the culture mixture to a concentration of 0.5 mM, followed by a further incubation period of 8 h to facilitate protein expression. For protein purification, Ni–NTA chromatography, provided by Beyotime Biotechnology, was used to lyse the 6 × His tags incorporated in the recombinant plasmids. The purification process included an initial phase of eluting nontarget proteins, followed by targeted elution of the desired proteins using a 300-mM imidazole solution. The sequences of primers used for constructing the pET28a::*srtA* recombinant plasmid and its mutant variants can be found in Table 1.

**Table 1:** Oligonucleotide primers used in this study

Primers	Nucleotide sequence (5′ → 3′)	Purpose
*SrtA*	GGGAATTCCATATGCAAGCTAAACCTCAAATTCCG	PCR
	CGCGGATCCTTATTTGACTTCTGTAGCTACAAAGA	
A92G	CCAGTATATCCAGGACCAGGCACACCTGAACAATTAAATAG	PCR
	CTATTTAATTGTTCAGGTGTGCCTGGTCCTGGATATACTGG	
T191A	GATTACAATGAAAAGGCCGGCGTTTGGGAAAAAC	PCR
	GTTTTTCCCAAACGCCGGCCTTTTCATTGTAATC	
W194A	GAAAAGACAGGCGTTGCCGAAAAACGTAAAATC	PCR
	GATTTTACGTTTTTCGGCAACGCCTGTCTTTTC	
R197A	GTTTGGGAAAAAGCCAAAATCTTTGTAG	PCR
	CTACAAAGATTTTGGCTTTTTCCCAAAC	

### Screening of SrtA inhibitors

To investigate the activity of the SrtA transpeptidase, we applied the FRET technique, as described in earlier research. A mixture of 100 μL of the SrtA protein at a concentration of 10 μM was combined with different concentrations of verbascoside in a dark environment at 37 °C for 1 h. After incubation, we added the fluorescent substrate peptide Abz-LPATG-Dap (Dnp)-NH_2_ at 10 μM. After 20 additional minutes of incubation at the same temperature, the fluorescence was measured using a Thermo microplate reader. Salvianolic acid A (Mu et al. 2020) was validated as a potent in vitro inhibitor of SrtA activity and served as a positive control. The SrtA protein itself was utilized as a negative control to establish baseline activity levels. We then calculated the inhibition rate of each compound, considering any compound with more than 70% inhibition as a probable inhibitor. After this, we plotted a concentration-dependent inhibition curve and determined the IC_50_ values for further investigation.

### Antibacterial activity assessment

The antibacterial activity of verbascoside against *S. aureus* USA300 was meticulously evaluated by determining its MIC via the National Comprehensive Cancer Study (NCCLS)–endorsed broth dilution methodology. This process involved the precise dispensing of 100 μL of cation-adjusted Mueller–Hinton broth (CAMHB) medium supplemented with 10^5^ CFUs of *S. aureus* USA300 into each well of a 96-well plate. A range of verbascoside concentrations, ranging from 16 to 1024 μg/mL, was administered via the double-dilution technique. Subsequently, each well was supplemented with 2.5 μL of resazurin solution (5 mg/mL). A control group devoid of treatment and containing only *S. aureus* USA300, along with a blank control composed solely of CAMHB medium, was also established. Furthermore, 3 μg/mL vancomycin served as the positive control. After a consistent incubation period of 16 h at 37 °C, the assessment of antibacterial efficacy was conducted visually by observing color shifts. The MICs of verbascoside against strains Newman, SA1B3B, and SA37 were subsequently assessed utilizing identical methodologies.

### Growth curve analysis

Growth curve analysis was conducted using overnight cultures of *S. aureus* USA300. After dilution at a ratio of 1:100, the cultures were inoculated into fresh TSB media and nurtured until they reached an OD_600_ of 0.3. The bacterial cultures were then divided into three groups: a solvent control group (WT + DMSO), a drug group (WT + verbascoside (64 μg/mL)), and a *ΔsrtA* group. Bacterial culture samples were collected at different time points to assess the absorbance at 600 nm, providing insights into the impact of verbascoside on bacterial growth dynamics.

### Cell viability assessment

A series of diluted A549 cells (human pulmonary epithelial cells) at a concentration of 2 × 10^4^ were meticulously inoculated into 96-well plates and incubated in a cell incubator for 24 h. Next, various concentrations of verbascoside were mixed, and the cells were incubated for an additional 24 h. Subsequently, 10 μL of CCK8 reagent (US EVERBRIGHT, Suzhou, China) was added, and after a 4-h incubation in the cell incubator, the absorbance was recorded at 450 nm utilizing a microplate reader.

### Assessment of the impact of verbascoside on *S. aureus* adhesion

Verbascoside was introduced into the *S. aureus* culture at an OD_600_ of 0.3, during which continuous growth was fostered until an OD_600_ of 0.5 was reached. The bacterial population was then harvested through centrifugation and subsequently resuspended in PBS. The resuspended bacteria were subsequently inoculated into 96-well plates precoated with 100 μL of a coating solution (20 μg/mL bovine blood fibrinogen). Cultivation ensued at 37 °C for 2 h, after which unbound bacteria were delicately eliminated through washing with PBS, and the adhered bacteria were fixed using 25% (v/v) formaldehyde for 30 min. Subsequent staining with crystal violet dye for 20 min preceded the absorbance measurement at 570 nm, providing insights into the modulation of bacterial adhesion influenced by verbascoside.

### Modulation of *S. aureus* USA300 biofilm formation by verbascoside

Diverse concentrations of verbascoside were initially introduced into *S. aureus* USA300, with an OD_600_ nm of 0.1. The bacterial culture was allowed to proliferate until an OD_600_ nm of 0.6 was reached at 37 °C. A minute aliquot (5 µL) of the bacterial culture was amalgamated with 200 µL of BHI broth and allowed to incubate for 18 h to foster robust biofilm formation. Subsequently, the medium was meticulously discarded, and the cell plate was thoroughly washed to eliminate residual bacteria. To assess biofilm formation, the samples were subjected to staining with 0.1% crystal violet for 20 min. After staining, the unbound dye was removed by rinsing with sterile PBS and air-drying at room temperature. Thereafter, 95% ethanol (100 µL) was added, and the absorption was quantified at 570 nm. This meticulous approach provided a comprehensive evaluation of the impact of varying verbascoside concentrations on the intricate process of *S. aureus* USA300 biofilm formation. Moreover, the formation of biofilms in strains Newman, SA1B3B, and SA37 was subsequently assessed following treatment with verbascoside, using consistent methodologies.

### Quantification of SpA levels in *S. aureus* USA300 treated with verbascoside

Overnight cultures of *S. aureus* USA300 and Δ*srtA* were diluted in TSB medium at a ratio of 1:1000. Subsequently, various concentrations of verbascoside (64 μg/mL or DMSO) were introduced to the respective cultures. Cultivation ensued at 37 °C until the OD_600_ reached 1.0, at which point the bacterial cultures were harvested through centrifugation and subjected to two washes with PBS. The bacteria were resuspended in 50 μL of FITC-labeled rabbit IgG (1:200), and the mixture was incubated in the dark for 2 h. After incubation, the bacteria were resuspended in 4% formaldehyde following a thorough wash. The ensuing fluorescence intensity, indicative of the quantity of SpA, was quantified using flow cytometry (Beckman Coulter, USA).

### Impact of verbascoside on the A549-*S. aureus* USA300 interaction

A549 cells were seeded at a density of 3 × 10^5^ cells per well and were meticulously inoculated into 24-well cell culture plates. Following an overnight incubation in an atmosphere containing 5% CO_2_ at 37 °C, the cells were primed for subsequent experimentation. Various concentrations of verbascoside (0–64 μg/mL) were combined with *S. aureus* USA300 and cultured until they reached an OD_600_ of 1.0. The bacterial population was then harvested through centrifugation and resuspended in an equivalent volume of DMEM. Subsequently, 1 mL of the resuspended bacterial solution was incubated in the cell medium for 2 h at 37 °C. After two washes with PBS, 1 mL of DMEM containing gentamicin (300 μg/mL) was added to the cell culture plates. Following a 1-h culture at 37 °C, the cells were washed with PBS. Subsequent cell lysis was facilitated by the addition of Triton X-100 to the cell culture plates. An appropriate aliquot of the lysate was then coated on TSB medium until the emergence of a single colony, which was subsequently enumerated.

### Quantification of SrtA expression in verbascoside-treated *S. aureus* USA300

*S. aureus* USA300 was subjected to various concentrations of verbascoside (0–64 μg/mL) and meticulously collected by centrifugation and subsequent resuspension in PBS. Total protein extraction from *S. aureus* was conducted using the conventional methodology as outlined previously. After extraction, an equivalent amount of total bacterial protein (20 μg/μL, 10 μL) was isolated and transferred onto a polyvinylidene fluoride membrane. The membrane was subsequently blocked with 5% BSA for 2 h. Subsequently, the sections were incubated with a rabbit anti-SrtA polyclonal antibody. Following a series of washing steps, the membrane was exposed to a horseradish peroxidase (HRP)–labeled goat anti-rabbit antibody for an additional 1 h. The resulting bands were meticulously exposed and detected, and the target band was subjected to comprehensive analysis. This methodology enabled the accurate assessment of SrtA expression levels in *S. aureus* USA300 under the influence of various concentrations of verbascoside.

### Reversible inhibition assay

The procedure for the reversible reaction was conducted in strict accordance with standard methods. To summarize, we combined purified SrtA protein with verbascoside in 100 μL of reaction buffer at a concentration 10 times greater than the IC_50_ and incubated this mixture for 1 h at 37 °C in the dark. For the control group, we used an equivalent amount of DMSO instead of verbascoside. After this, the reaction mixture was diluted 100 times, after which the peptide substrate Abz-LPATG-Dap (Dnp)-NH_2_ was added, followed by another 20 min of incubation at 37 °C. We then measured the fluorescence intensity using a microplate reader at specified wavelengths (420 nm for emission and 309 nm for excitation). Based on these readings, we calculated the rate of reversible inhibition. A rate above 60% was indicative of noncovalent reversible binding, while a rate below 60% indicated covalent irreversible binding.

### Determination of the binding constants between verbascoside

To assess the binding affinity, the binding constant (*K*_*A*_) between verbascoside and SrtA was quantified. This was achieved through a fluorescence quenching assay, as described in previous studies (Papadopoulou et al. 2005). The fluorescence emission spectra of SrtA were recorded in the absence and presence of various concentrations of verbascoside. The excitation and emission wavelengths were carefully noted, with slit widths set at 5 and 10 nm, respectively. The resulting fluorescence quenching data were plotted against different verbascoside concentrations, depicting the relative fluorescence intensity. The calculation of *K*_*A*_ values was performed utilizing established methods previously reported, providing valuable insights into the binding dynamics between verbascoside and SrtA, as well as its distinct mutants.

### Structural analysis and molecular docking of SrtA with verbascoside

The structural basis of SrtA was elucidated through the extraction of its three-dimensional X-ray crystal structure (PDB ID: 1T2P) from the Protein Data Bank. Similarly, the three-dimensional structures of verbascoside were meticulously constructed using ABEE. Subsequent molecular docking studies between the SrtA protein and verbascoside were performed according to standard procedures. AutoDock-Tools 1a.5.6 software (La Jolla, CA, USA) was used to conduct the docking analysis, shedding light on the potential interactions and binding configurations between SrtA and verbascoside. This structural elucidation provides crucial insights into the molecular interplay governing the interaction between SrtA and verbascoside.

### Evaluation of the acute toxicity of verbascoside in *Galleria mellonella*

*Galleria mellonella* larvae were utilized to assess the toxicity of verbascoside. The larvae were randomly divided into four groups, each consisting of ten individuals. Two experimental groups received postventral foot injections of verbascoside at concentrations of 20 mg/kg and 40 mg/kg. Two positive control groups were administered an equivalent volume of PBS and left untreated. Following the injections, an extensive observation period of five days ensued, during which all *Galleria mellonella* larvae were closely monitored for signs such as blackening, sluggish movement, or mortality.

### Evaluation of the acute toxicity of verbascoside in mice

The acute toxicological profile of verbascoside was investigated in female C57BL/6 J mice aged 6–8 weeks. The study group, comprising twenty-four mice, was randomly divided into four groups. Two experimental groups of mice were injected subcutaneously with verbascoside at concentrations of 20 mg/kg and 40 mg/kg. The control group received an equivalent volume of PBS. Additionally, an untreated group served as the positive control in this study. Subsequently, an extensive observation period of 7 days followed, during which all mice were closely monitored for changes in spontaneous movement and for the occurrence of abnormal behaviors or fatal outcomes.

### Evaluating the efficacy of verbascoside against *S. aureus* in the *Galleria mellonella* infection model

With respect to the *Galleria mellonella* infection model, we investigated the effectiveness of verbascoside against *S. aureus*. *Galleria mellonella* larvae were divided into four groups: a USA300 infection cohort, an untreated control group, and verbascoside-treated groups (40 mg/kg), with Δ*srtA* each comprising 10 larvae. To induce infection, larvae were injected with a 10-μL suspension of MRSA (5 × 10^6^ CFU/mL) in the leftmost proleg. Treatment commenced 1-h postinoculation, with designated doses administered to the Verbascoside groups (40 mg/kg), while the control group remained untreated. Larvae were maintained in a constant 37 °C environment, and their survival was monitored every 12 h for 120 h.

Concurrently, for colony count assessments, methodologies akin to those used in the survival study were employed. Larvae were harvested 48 h postinfection, subjected to sterilization, homogenized, and cultured on TSA at 37 °C for 24 h to quantify the bacterial colonies. This approach established a sensitivity threshold of 100 CFU/mL for larval homogenates. Additionally, we established infection groups of *Galleria mellonella* using only the Δ*srtA* mutant, followed by treatment with verbascoside after inoculation. These groups were utilized to monitor the survival rates and bacterial counts in *Galleria mellonella*, aiding in the analysis of verbascoside’s in vivo targeting effects. All experiments were replicated a minimum of three times to ensure the robustness and reliability of the findings.

### In vivo assessment of verbascoside in treating MRSA-induced acute pneumonia

To investigate the therapeutic efficacy of verbascoside against acute pneumonia caused by MRSA, a pneumonia infection model was established in female C57BL/6 J mice aged 6–8 weeks. Mice were randomized into four distinct cohorts: the MRSA USA300 infection group (WT), Δ*srtA* infection group, verbascoside treatment group, and a noninfected control. Δ*srtA* and WT + DMSO served as positive and negative controls, respectively. For survival experiments, each group encompasses ten mice. The mice were intranasally infected with 2 × 10^8^ CFU of *S. aureus* USA300, ensuring proper aspiration of bacteria into the lungs by holding them upright for 30 s. Subsequently, verbascoside was administered subcutaneously at a dose of 40 mg/kg beginning 1 h postinfection, and the administration of verbascoside was repeated every 12 h. The survival rate of the mice was meticulously recorded at 12-h intervals over a 96-h period.

To assess the therapeutic impact of verbascoside, the bacterial load in the lung tissue was quantified, and histopathological changes in the lungs were examined (*n* = 6). Following a two-day intranasal infection period with 30 μL (1 × 10^8^ CFU) of *S. aureus* culture, the mice were euthanized, and lung samples were collected, weighed, and homogenized. The homogenate was appropriately diluted and plated on BHI agar, and after overnight incubation at 37 °C, colony quantification was performed. The left lungs were fixed with 10% formalin after perfusion, and hematoxylin and eosin (H&E) or F4/80 staining was conducted to observe and record pathological changes in the lung tissue. Furthermore, we established a pneumonia model in mice using the Δ*srtA* alone, followed by verbascoside treatment postinoculation with the Δ*srtA*. These groups were employed to observe pneumonia survival rates and lung dry/wet weight ratios.

### Statistical analysis

The data are expressed as the mean ± SEM for each group in the individual experiments. The experimental data in this study were analyzed using GraphPad Prism 8.0. *p* values < 0.05 were considered to indicate statistical significance.

## Results

### Identification of verbascoside as a potent inhibitor of SrtA

**Fig. 1:**
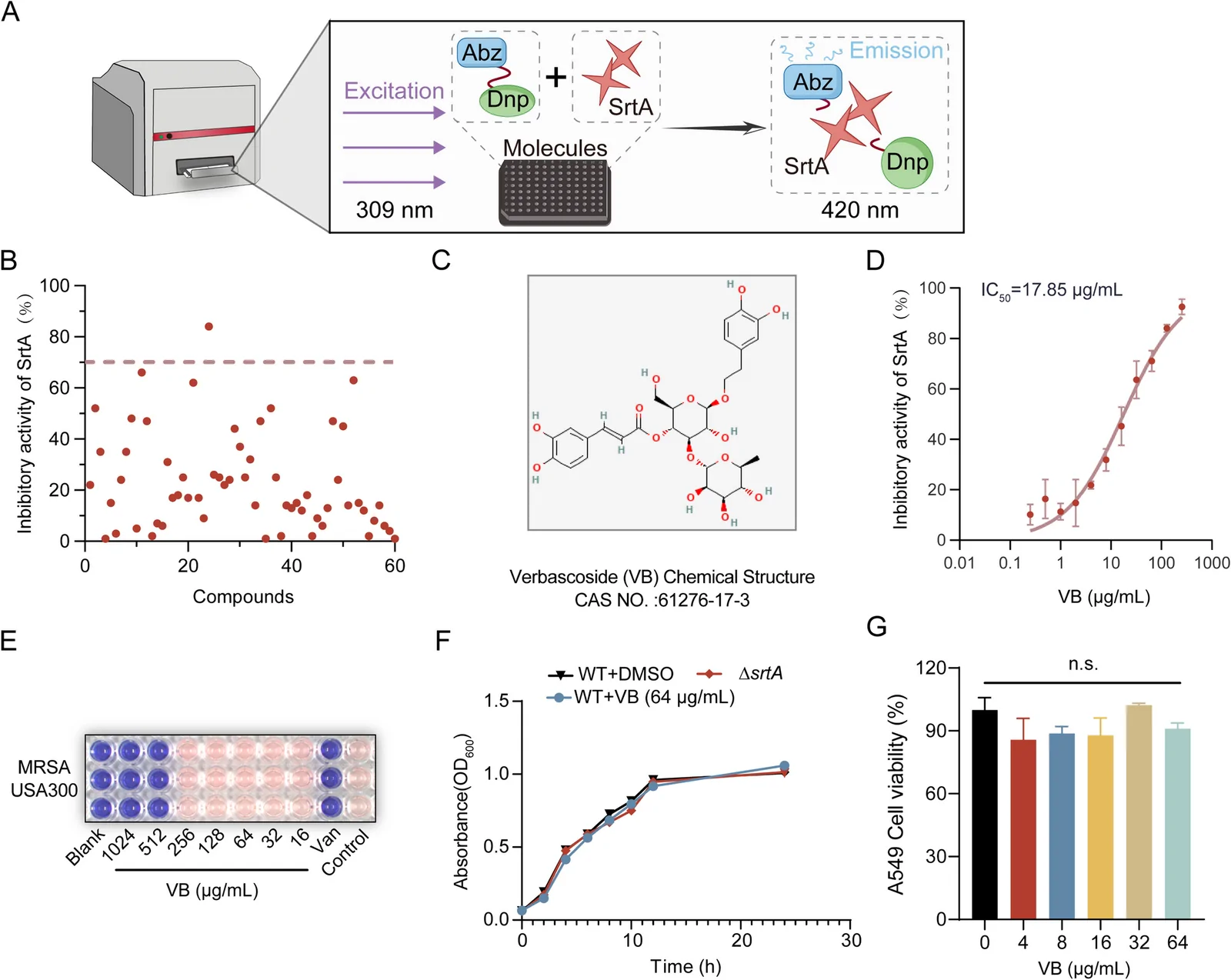
Verbascoside suppresses SrtA enzyme activity. **A** A schematic representation outlines the screening method for inhibitors, focusing on the SrtA-mediated cleavage of the Abz-LPATG-Dap (Dnp)-NH_2_ substrate, which triggers an energy shift and alters fluorescence. **B** The effectiveness of various compounds in inhibiting SrtA was explored. Compounds showing more than 70% inhibition were identified as having potential inhibitory effects. **C** The chemical structure of verbascoside is presented, providing a detailed molecular perspective. **D** Verbascoside inhibited the activity of SrtA in a dose-dependent manner on the Abz-LPATG-Dap (Dnp)-NH_2_ substrate, with an IC_50_ value established at 17.85 μg/mL. **E** The MIC of verbascoside against the USA300 strain is displayed, indicating that it has no antibacterial efficacy. **F** Growth curves for *S. aureus* USA 300 and the Δ*srtA* mutant strain treated with and without verbascoside at a concentration of 64 μg/mL to determine its impact on bacterial proliferation. **G** The percentages of viable A549 cells after 24 h of treatment with verbascoside at concentrations ranging from 0 to 64 μg/mL were determined using the CCK-8 method

We initiated the identification of SrtA inhibitors from a natural compound library utilizing fluorescence resonance energy transfer (FRET) as our screening method. The fundamental premise underlying SrtA screening lies in the capacity of the SrtA protein to recognize and cleave the fluorescent substrate peptide Abz-LPATG-Dap (Dnp)-NH_2_, thereby eliciting a measurable fluorescence shift (Fig. 1A). Prior to the formal screening of SrtA inhibitors, the isolation of the SrtA protein was achieved through initial Ni column purification (Fig. S1). Our screening endeavor involved the examination of 60 natural compounds, ultimately pinpointing one specific compound—verbascoside (Fig. 1B–C). Remarkably, verbascoside emerged as a potent inhibitor of SrtA activity, exhibiting an IC_50_ of 17.85 µg/mL (Fig. 1D).

Subsequently, the antimicrobial efficacy of verbascoside against *S. aureus* USA300 was meticulously evaluated. The determined minimum inhibitory concentration (MIC) of verbascoside against USA300 was 512 μg/mL (Fig. 1E). Similarly, the growth curve data at 64 μg/mL suggested that verbascoside had a negligible influence on the proliferation of USA300 (Fig. 1F). Furthermore, the MICs of verbascoside against other strains, namely, Newman, SA1B3B, and SA37, were consistently recorded at 1024 μg/mL each, as detailed in Figure S3, highlighting its limited efficacy against *S. aureus*.

Moreover, to ascertain its cytotoxic potential, MTT assays were conducted on the A549 cell line, revealing no significant deviation in cell viability across groups. This observation suggested that even at concentrations threefold greater than the IC_50_, verbascoside exhibited minimal cytotoxicity (Fig. 1G). Subsequent assessments involving red blood cell lysis experiments, as well as evaluations on *Galleria mellonella* and mice, aimed at further delineating the safety profile of verbascoside. Encouragingly, at a concentration of 64 µg/mL, verbascoside did not induce erythrocyte rupture in rabbit red blood cells (Fig. S2).

Additionally, when *Galleria mellonella* larvae were subjected to injections of 20 mg/kg or 40 mg/kg verbascoside, they exhibited no distress signals, such as discoloration, sluggish movement, or mortality, within a 5-day observation period (Table 2). Similarly, mice administered 20 mg/kg or 40 mg/kg verbascoside displayed no abnormal behavioral patterns or fatal outcomes. Collectively, these findings substantiate the assertion that the tested concentrations of verbascoside are devoid of acute in vivo toxicity (Tab. S1). Consequently, verbascoside has emerged as a promising small-molecule inhibitor of SrtA, offering substantial inhibitory effects without significant toxicity.

**Table 2:** Survival rates of the *Galleria mellonella* larvae

Groups	Larvae survival (%)				
	Day 1	Day2	Day 3	Day 4	Day 5
Noninjected	100	100	100	100	100
Control (PBS)	100	100	100	100	100
Verbascoside 20 mg/kg	100	100	100	100	100
Verbascoside 40 mg/kg	100	100	100	100	100

### Impact of verbascoside on the pathogenicity of *S. aureus*

**Fig. 2:**
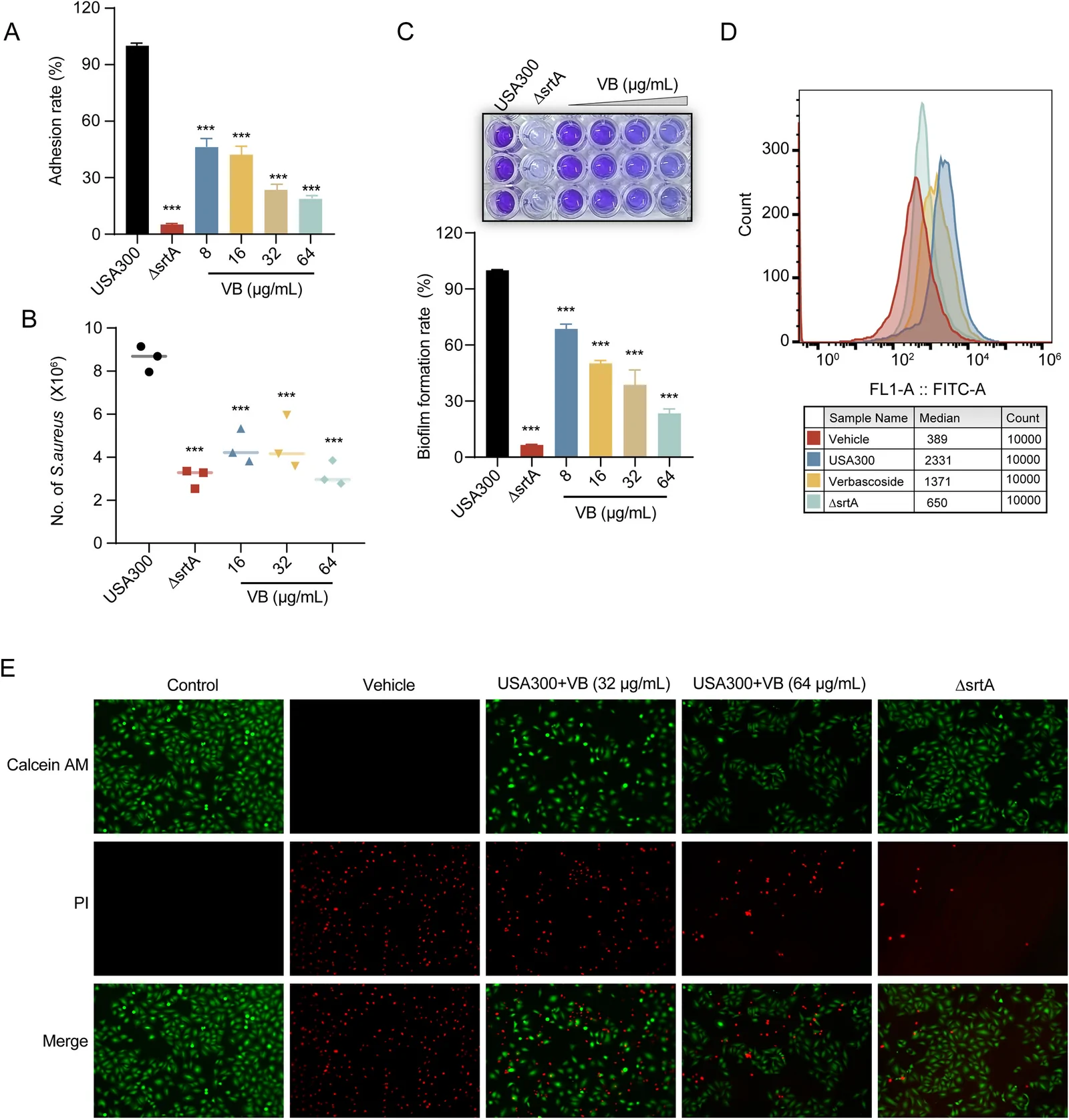
Verbascoside modulates *S. aureus* virulence traits via SrtA. **A** Impact of verbascoside on the adherence of *S. aureus* USA300 to fibrinogen. **B** Inhibition of *S. aureus* USA300 internalization into A549 cells by verbascoside. **C** Quantitative assessment of *S. aureus* biofilm formation under verbascoside exposure using crystal violet staining. **D** Examination of SpA expression on *S. aureus* through flow cytometry employing FITC-conjugated rabbit IgG, with the Δ*srtA* variant serving as a reference. **E** Verbascoside-mediated attenuation of MRSA virulence toward A549 cells, as evidenced by live-dead cell staining

The biological effects of verbascoside on SrtA were comprehensively evaluated through a series of experiments. Initially, the focus was on assessing the impact of verbascoside on the adhesion of *S. aureus* to fibrinogen, a critical process mediated by SrtA and pivotal for bacterial pathogenicity. Notably, in the absence of fibrinogen-binding proteins, bacterial adhesion to host tissue diminishes. Treatment with verbascoside (64 µg/mL) significantly reduced *S. aureus* adhesion to fibrinogen to 19.89 ± 1.43% of that in the USA300 group (Fig. 2A).

Given the intricate relationship between *S. aureus* SrtA and host colonization, along with the pathogenesis of invasive diseases, we further investigated the invasive potential of verbascoside-treated *S. aureus* on A549 cells. The infection of A549 cells by verbascoside-treated *S. aureus* resulted in a notable reduction in the presence of bacteria within the cells compared to that in the USA300 group (*p* < 0.001), indicating the ability of verbascoside to attenuate *S. aureus* invasion of A549 cells by targeting SrtA (Fig. 2B).

With respect to biofilm formation, a crystal violet staining assay provided insights into the role of SrtA in this process. Here, the biofilm biomass of Δ*srtA* was strikingly low, at 3.06 ± 0.45%, underscoring the significance of SrtA in biofilm formation. Verbascoside (64 µg/mL) emerged as a potent inhibitor of biofilm formation, even at a low dose of 8 µg/mL, compared to that in the USA300 group (*p* < 0.001, Fig. 2C). Impressively, this inhibitory effect extended to biofilm formation in both the Newman strain and clinical isolates SA1B3B and SA37 (Fig. S4).

Further investigation of the virulence factor surface protein A (SpA) revealed its crucial role in immune evasion. Specifically, the binding of SpA to the Fc region of IgG enhances immune evasion by impeding opsonophagocyte clearance. The measurement of IgG fluorescence intensity via flow cytometry revealed the impact of verbascoside on SpA levels. The diminished fluorescence intensity in the Δ*srtA* group suggested impaired SpA anchoring, with a similar reduction observed in the verbascoside treatment group compared to the USA300 group (*p* < 0.001, Fig. 2D).

Subsequent studies aimed to ascertain the potential protective role of verbascoside against MRSA infection in A549 cells. Notably, the live/dead cell assay revealed minimal killing effect exerted by the Δ*srtA* on A549 cells compared to the USA300 group. Additionally, the ability of verbascoside-treated *S. aureus* to kill A549 cells was significantly attenuated (Fig. 2E).

In summary, these findings underscore the ability of verbascoside to inhibit SrtA-associated virulence phenotypes, including the adhesion, invasion, and biofilm formation of MRSA in vitro.

### Direct interaction between verbascoside and SrtA

**Fig. 3:**
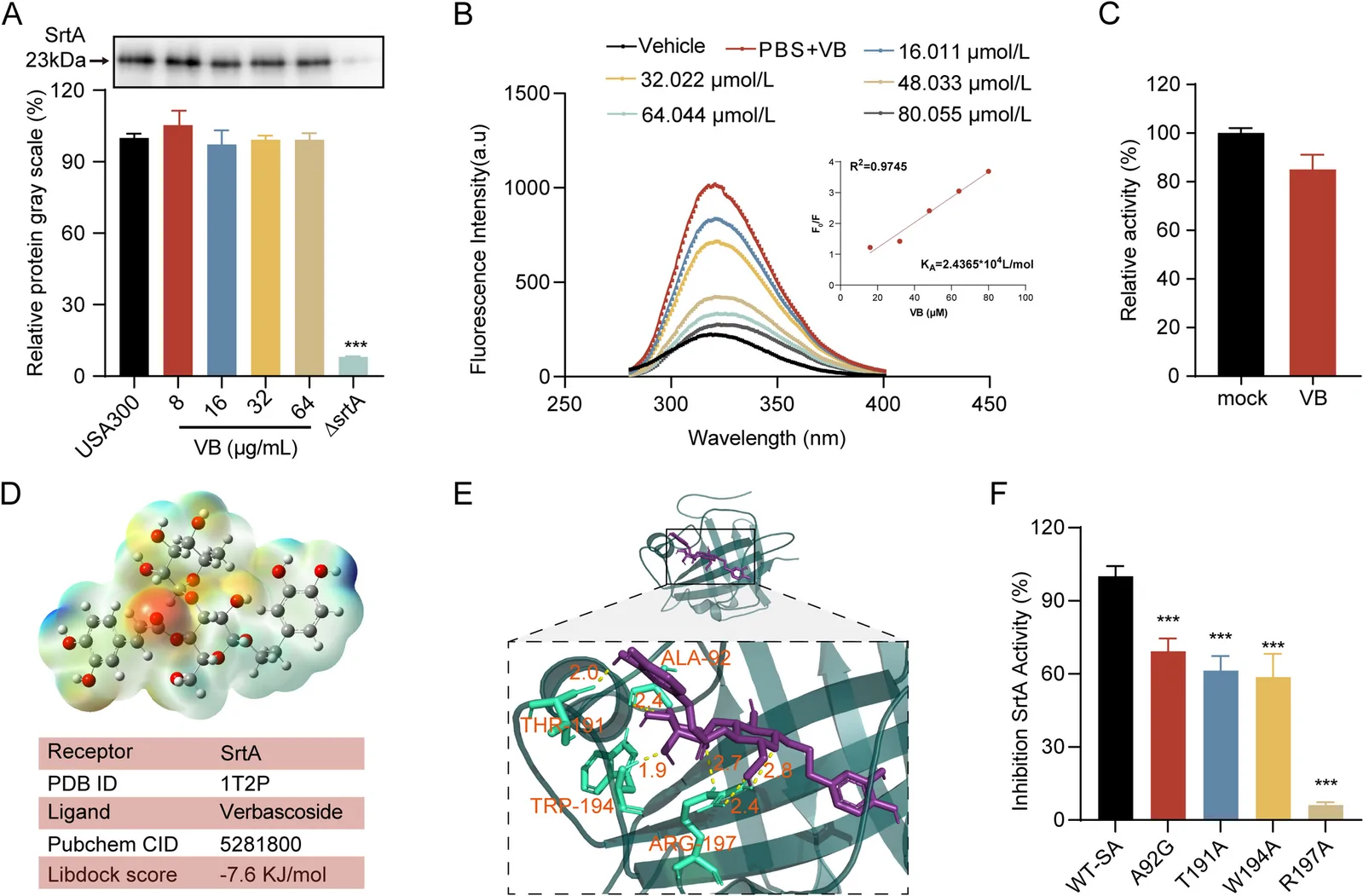
Direct interaction of verbascoside with SrtA. **A** Western blot of SrtA from *S. aureus* exposed to various concentrations of verbascoside (0 to 64 μg/mL). **B** Examination of the interaction between verbascoside and SrtA using the fluorescence quenching method, revealing a gradual decrease in SrtA fluorescence with increasing verbascoside concentration. **C** FRET assay analysis of SrtA activity after treatment with 10 × IC_50_ verbascoside, with untreated SrtA (mock) set to 100%. **D** Three-dimensional electrostatic potential mapping of verbascoside visualized via ABEE. **E** Molecular docking plot illustrating the interaction dynamics between verbascoside and SrtA, indicating a total binding free energy of − 7.6 kcal/mol. **F** Evaluation of the impact of verbascoside on the enzymatic activity of SrtA and its variants (A92G, T191A, W194A, and R197A) via FRET

To further elucidate how verbascoside modulates the action of SrtA, Western blot analysis was initially conducted to determine its potential interference with SrtA expression. Remarkably, the expression levels of the SrtA protein remained consistent across various concentrations of verbascoside (ranging from 0 to 64 µg/mL) and were comparable to those in both the USA300 and Δ*srtA* groups. This observation strongly suggested that verbascoside did not modulate SrtA expression (Fig. 3A).

Subsequent fluorescence quenching assays revealed a concentration-dependent reduction in the fluorescence intensity of SrtA upon exposure to increasing concentrations of verbascoside. This quenching phenomenon, depicted through the linear relationship of F0/F with the quencher concentration, facilitated the construction of a Stern–Volmer plot, yielding a calculated binding constant (*K*_*A*_) of 2.4365 × 10^4^ L/mol. This indicated a significant interaction between verbascoside and SrtA (Fig. 3B).

To ascertain the reversibility of SrtA inhibition by verbascoside, a mixture of SrtA with a tenfold increase in the IC_50_ of verbascoside was cultured with fluorescent peptide substrates. Remarkably, the recovery rate reached 85.09% compared to that of the untreated SrtA group, indicating that verbascoside functions as a reversible inhibitor of SrtA by binding noncovalently to its active site (Fig. 3C).

Subsequently, electrostatic potential mapping of the molecular surface was conducted to identify the optimal docking orientations and interaction sites for molecular docking simulations (Fig. 3D). These simulations revealed that verbascoside binds primarily to the binding pocket of SrtA through hydrogen bonding and electrostatic interactions. Detailed analysis unveiled six crucial hydrogen bonds formed between the side chains of key residues in SrtA and verbascoside, resulting in a total binding free energy of − 7.6 kJ/mol (Fig. 3D, E).

Further investigations involving point mutations in key amino acid sites (ALA-92, THR-191, TRP-194, and ARG-197) revealed purified mutant SrtA proteins. Through FRET analysis, the impact of verbascoside on the transpeptidase activity of these mutant SrtA proteins was assessed. Notably, significant reductions in transpeptidase inhibitory capacity were observed for the mutant SrtA proteins (*p* < 0.001, Fig. 3F). Mutation at the R197 site nearly abolished transpeptidase activity, consistent with prior studies (Frankel et al. 2007). In essence, the amalgamation of molecular docking and point mutation assays confirmed the critical role of ALA-92, THR-191, TRP-194, and ARG-197 as pivotal amino acid sites for verbascoside binding to SrtA.

### Assessment of the toxicity and protective effects of verbascoside in a *Galleria mellonella* model against *S. aureus* infection

*Galleria mellonella* larvae provide a valuable alternative model for assessing the in vivo toxicity and efficacy of novel antimicrobial agents, offering a rapid and cost-effective premammalian host experimental setup (Cé et al. 2020). This study investigated the impact of verbascoside on *Galleria mellonella* larvae, focusing on its toxicity and protective effects against MRSA USA300.

**Fig. 4:**
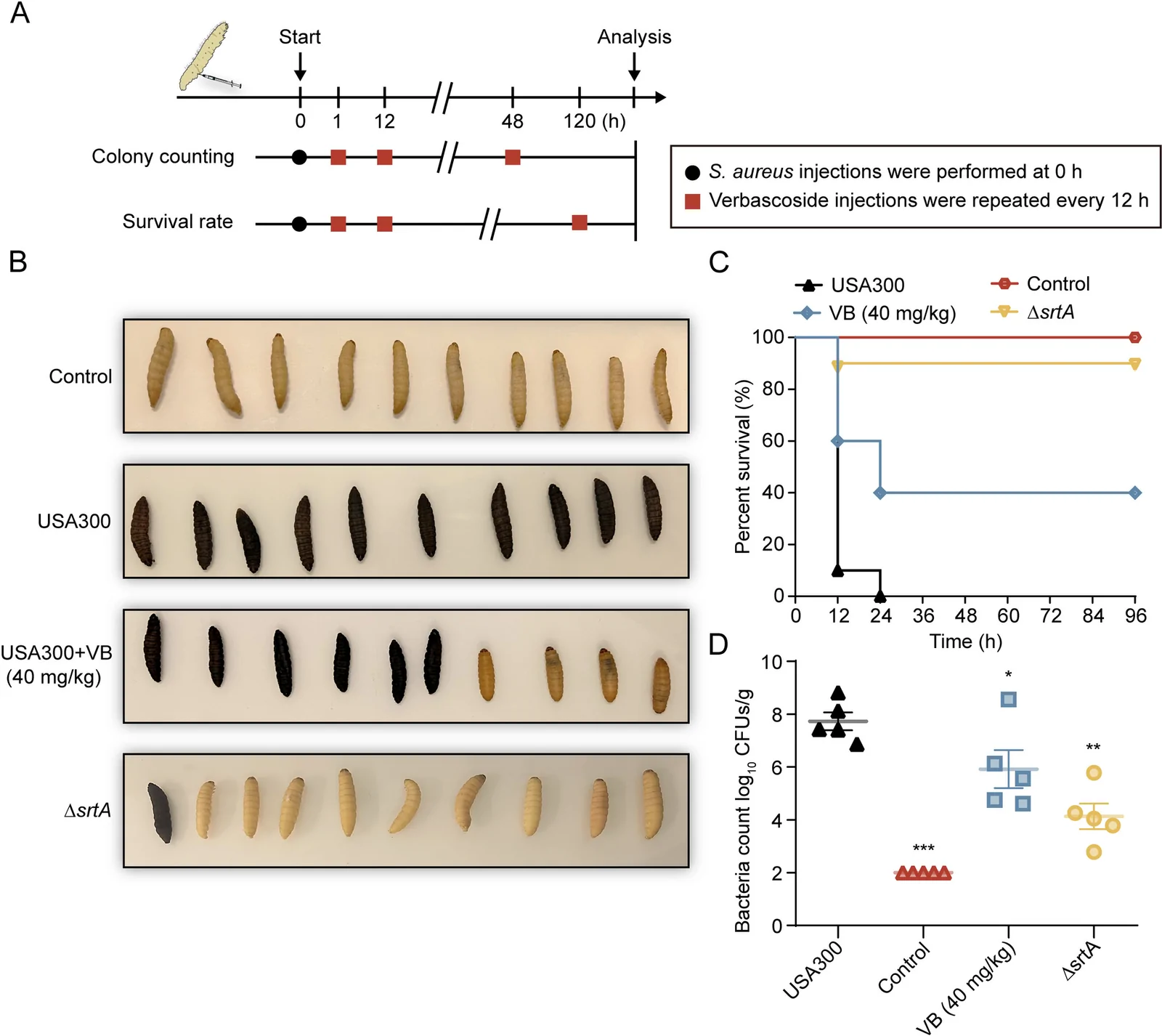
Verbascoside increased MRSA *Galleria mellonella* survival. **A** Visual representation of the progression and evaluation of MRSA infection in *Galleria mellonella* cell model. **B**, **C** A comparative investigation revealed distinct *Galleria mellonella* cohorts (each consisting of 10 larvae), including an untreated control group, an *S. aureus* USA300-infected group, a Δ*srtA*-infected group, and groups administered verbascoside (40 mg/kg). The survival rates of these cohorts were documented at 120 h postinfection. **D** Enumeration of colony-forming units (CFUs) in *Galleria mellonella* larvae following infection (*n* = 5), determined through the agar dilution method

*Galleria mellonella* larvae were intrahemocoelically infected with USA300 and the *ΔsrtA*. Safety experiments were conducted using various concentrations of verbascoside, and the efficacy of verbascoside in treating *S. aureus* infection was also assessed. Over the subsequent 120-h period, or a condensed 48-h period, larval survival, appearance, pigmentation, and bacterial burden were monitored (Fig. 4A).

Survival curves revealed a significant decrease in larval survival following MRSA infection, plummeting to 0%, accompanied by a decrease in body color indicative of severe infection. However, treatment with verbascoside (40 mg/kg) resulted in a notable increase in larval survival, reaching 40% (Fig. 4B, C). Furthermore, the bacterial load in the verbascoside-treated group significantly decreased from a baseline of 7.729 to 5.920 (Fig. 4D). Furthermore, we employed an MRSA-infected large wax moth model to evaluate the survival and bacterial load outcomes of *G. mellonella* inoculated with either the SrtA deletion strain alone or in treatment with verbascoside. The results revealed no significant disparity between the two groups, confirming the targeted action of verbascoside against SrtA and its consequent effectiveness in mitigating MRSA pathogenicity in vivo (Fig. S5). This finding underscores the substantial protective effect of verbascoside on *Galleria mellonella* during infection, demonstrating its promising therapeutic potential.

### Therapeutic efficacy of verbascoside in treating MRSA-induced pneumonia

**Fig. 5:**
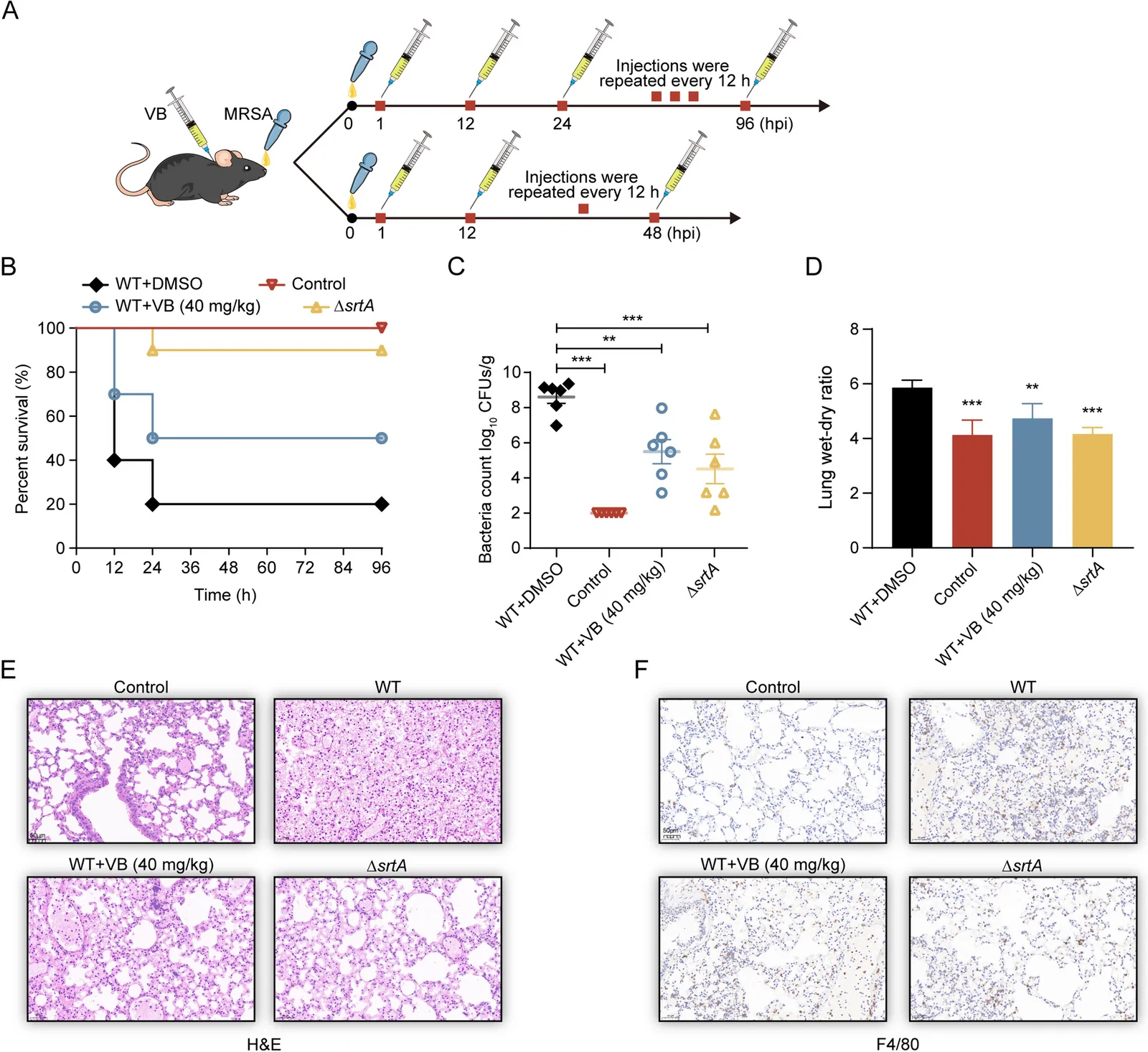
Treatment of mice with verbascoside-induced *S. aureus*–induced pneumonia. **A** Prediction of the establishment of a pneumonia model in mice through nasal administration of MRSA, followed by an evaluation of survival rates and pathological alterations. **B** Impact of verbascoside on the survival rate of mice (*n* = 10) exposed to a lethal dose of *S. aureus*, revealing a significant increase in survival in the verbascoside-treated group compared to the wild-type (WT) group. **C** The reduction in the bacterial load in the lungs of mice (*n* = 6) treated with verbascoside at a dose of 40 mg/kg was exaggerated, demonstrating a notable decrease in the bacterial load in the verbascoside group relative to that in the WT group. **D** Assessment of the lung dry-to-wet weight ratio as an indicator of the therapeutic efficacy of verbascoside in MRSA-induced pneumonia in mice. **E**, **F** Elaboration of microscopic lung alterations in mice by H&E or F4/80 staining, contrasting those treated with verbascoside (40 mg/kg) with those in the control group, accompanied by histopathological lung examinations. The scale bar represents 50 μm

MRSA-induced bacterial pneumonia poses a significant infectious challenge in both community and hospital settings due to its complexity, high complication rate, and mortality. Therefore, the clinical management of such infections is complex. To assess the impact of verbascoside on MRSA infection, normal mice were intranasally infected with lethal doses (2 × 10^8^ CFU) of *S. aureus* USA300, and their survival rates were monitored every 12 h for 96 h Additionally, another group of normal mice was intranasally infected with sublethal doses (1 × 10^8^ CFU) of *S. aureus* USA300 for 48 h to further evaluate the effects of verbascoside on MRSA pneumonia. The impacts on pulmonary bacterial load, the lung wet/dry weight ratio, and histopathological changes were assessed (Fig. 5A).

In the survival experiments, the survival rate of the USA300 group (lethal dose) decreased to 20%, while that of the Δ*srtA* group decreased to 90% at 96 h post infection, indicating the critical role of SrtA in the acute pneumonia model induced by *S. aureus*. Treatment with verbascoside (40 mg/kg) increased the survival rate of mice to 50%, indicating a protective effect of verbascoside on lung tissue in *S. aureus*–infected mice (Fig. 5B). Subsequent evaluations revealed a significant decrease in the verbascoside-treated group, from 8.609 to 5.491 log CFU/g (Fig. 5C), indicating reduced *S. aureus* invasion and consequent lung damage compared to those in the USA300 group. The lung wet/dry weight ratio confirmed a decrease in pulmonary edema and tissue injury posttreatment (Fig. 5D). Histopathological examination of the lungs of the USA300 group mice revealed marked congestion and extensive inflammatory cell accumulation in the alveolar spaces. However, after treatment with verbascoside, a substantial reduction in inflammatory cell infiltration was noted, with relatively intact alveolar structures (Fig. 5E). Immunohistochemical staining for F4/80 confirmed a significant decrease in macrophage infiltration (Fig. 5F), further supporting the therapeutic potential of verbascoside in alleviating the severity of MRSA-induced pneumonia in mice.

Additionally, we utilized a pneumonia infection model to assess the survival and lung wet/dry ratios of mice inoculated with the SrtA deletion strain alone or the SrtA deletion strain in treatment with verbascoside. The results revealed no discernible difference between the two groups, confirming verbascoside’s targeted action against SrtA and its efficacy in mitigating MRSA-induced pneumonia in vivo (Fig. S6). This in vivo study provides valuable insights into the potential therapeutic role of these compounds.

## Discussion

The antibiotic resistance of *S. aureus*, particularly its widespread resistance to β-lactam antibiotics, including penicillin and its derivatives, poses a global public health challenge (Guo et al. 2020). *S. aureus* has developed resistance to not only traditional antibiotics but also nearly all available antibiotics. In this context, vancomycin has become the last-resort antibiotic for treating MRSA infections. However, the emergence of vancomycin-resistant (VRSA) and VISA strains has further complicated treatment efforts (McGuinness et al. 2017). In addition to exhibiting specific antibiotic resistance, *S. aureus* exhibits nonspecific resistance through biofilm formation and plays a key role in many biofilm-associated infections (Hall and Mah 2017). Against this complex backdrop, developing alternative therapeutic strategies for bacterial infections is an urgent and challenging research direction.

Distinct from many bacteria, *S. aureus* does not rely solely on one or a few toxins to promote disease; rather, it produces a surprising array of virulence factors, including a multitude of toxins, immune evasion factors, and various protein and nonprotein factors, which collectively facilitate bacterial colonization during host infection (Cheung et al. 2021; Howden, Giulieri et al. 2023). Among these proteins, certain surface proteins with a C-terminal LPXTG motif are recognized by the membrane cysteine transpeptidase SrtA, a process crucial for anchoring proteins to the cell wall (Mazmanian et al. 1999; Gao et al. 2022, Ragab et al. 2023). SrtA has thus become an important drug target, particularly for controlling biofilm accumulation and adhesion proteins. Notably, SrtA is not essential for bacterial growth or viability.

In the search for SrtA inhibitors, FRET assays have been used to screen for inhibitors that recognize and cleave peptides with the LPXTG motif. During this process, verbascoside was identified as an effective SrtA inhibitor. Verbascoside is present not only in the *Verbascum* genus but also in more than 200 plant species. Verbascoside is a major component of L. verbena and has a range of biological activities, including anti-inflammatory, antioxidant, antitumor, and antibacterial effects (Nigro et al. 2020). Our research reveals the potential of verbascoside for combating MRSA virulence. As an antivirulence drug, verbascoside did not have significant antibacterial effects at concentrations far exceeding its IC_50_, suggesting its significant potential in preventing resistance. Additionally, verbascoside effectively inhibited *S. aureus* biofilm formation. Biofilm formation is a key step in *S. aureus* infection, particularly in the initial stages of adhering to various surfaces, and is critical for survival in food or invasion into host cells. In biofilms, a multitude of *Staphylococcal* surface proteins, including fibrinogen-binding proteins such as FnbpA, FnbpB, and ClfA, are mobilized for adhesion; these proteins are all targets of SrtA and are thus anchored to the cell wall. These findings indicate that verbascoside can disrupt biofilm formation by inhibiting SrtA.

SpA, a critical virulence factor in *S. aureus*, plays a multifaceted role in the pathogenesis of infections caused by this bacterium (Rigi et al. 2019). SpA is known primarily for its ability to bind the Fc region of immunoglobulin G (IgG), thereby interfering with host immune responses (Lindmark et al. 1983; Powers and Bubeck Wardenburg 2014). This binding impedes opsonization and phagocytosis, allowing *S. aureus* to evade the host immune system. Moreover, SpA has been implicated in biofilm formation, a key aspect of *S. aureus* infections, particularly in chronic and device-related infections. By promoting biofilm formation, SpA contributes to the ability of bacteria to adhere to surfaces and resist antimicrobial treatment, posing significant challenges in clinical settings. Adhesion and invasion are the initial stages at which a pathogen infects its host. Understanding the relationships among adhesion, invasion, and the host is essential (Niemann et al. 2004). We were pleased to find that verbascoside inhibits *S. aureus* adhesion to fibrinogen and reduces bacterial invasion of A549 cells, thereby protecting against MRSA damage. To further elucidate how verbascoside affects SrtA function, we first conducted Western blot analysis of the effect of verbascoside on SrtA expression. The results showed that verbascoside does not affect SrtA expression, suggesting that it may exert its effects by interfering with SrtA activity. Subsequent fluorescence quenching and molecular docking revealed a direct interaction between verbascoside and SrtA. Recent studies have suggested that SpA may also play a role in the invasion of epithelial and endothelial cells, potentially contributing to the dissemination of *S. aureus* within the host. In vivo experiments further validated our expectations, confirming the protective role of verbascoside in models of wax moth and pneumonia infections.

Research has highlighted the potential role of SpA in the penetration of epithelial and endothelial cells, potentially aiding the dissemination of *S. aureus* within the host (Gómez et al. 2004; Soong et al. 2011). Consequently, the observed protective effects in murine pneumonia models and *Galleria mellonella* (wax moth) infection assays may be intimately linked to the influence of SpA and other surface proteins regulated by SrtA. This connection underscores the complex interplay between bacterial virulence factors and host defense mechanisms, illuminating potential pathways for therapeutic intervention.

These findings not only deepen our understanding of the complex resistance and virulence mechanisms of *S. aureus* but also showcase the tremendous potential of using natural compounds such as verbascoside as potential treatment strategies. This new antivirulence approach, in particular by targeting virulence factors rather than through direct antibacterial action, offers new avenues for overcoming existing antibiotic resistance issues. Although further research is needed to validate and optimize these strategies, these preliminary results offer new hope for overcoming the challenge of antibiotic resistance.

## Supplementary Information

Below is the link to the electronic supplementary material.

Supplementary file1 (PDF 334 KB)

## Data Availability

The datasets generated during and/or analyzed during the current study are available from the corresponding authors upon reasonable request.
