# Supplementary material for: Unraveling the efficacy of verbascoside in thwarting MRSA pathogenicity by targeting sortase A
Source: Appl Microbiol Biotechnol. 2024 Jun 5;108:360. doi: 10.1007/s00253-024-13202-6 (PMC11153306; doi:10.1007/s00253-024-13202-6)
Supplement: Supplementary file 1 [file 253_2024_13202_MOESM1_ESM.pdf]

Supplementary material

Unraveling the Efficacy of Verbascoside in Thwarting MRSA Pathogenicity by Targeting Sortase A

Xingchen Li [1,#], Yingying Hou [2,#], Haoyan Zou [1], Yueying Wang [1], Yueshan Xu [1], Li Wang [1], Bingmei Wang [1], Ming Yan [1,*], Xiangyang Leng [1,*]

[1] Changchun University of Chinese Medicine, Changchun, China
[2] The First Affiliated Hospital of Zhengzhou University, Zhengzhou, China
[*] Correspondence:
Xiangyang Leng: lengxiangy@163.com
Ming Yan: mingyan.ccucm@outlook.com

[#] These authors contributed equally

Table of contents:

Supplementary Figure S1. Electrophoretic profiling of the effect of the purified SrtA protein via SDS–PAGE.
Supplementary Figure S2. The impact of different concentrations of verbascoside on RBC integrity.
Supplementary Figure S3. The MIC of verbascoside against standard and clinical strains of *S. aureus* stained with resazurin.
Supplementary Figure S4. Quantitative assessment of *S. aureus* biofilm formation under verbascoside exposure using crystal violet staining.
Supplementary Figure S5. Impact of verbascoside on the survival rate of *S. aureus* USA300 Δ*srtA* strain infected with *Galleria mellonella*.
Supplementary Figure S6. Treatment of mice with verbascoside-induced *S. aureus* USA300 Δ*srtA* strain-infected pneumonia.
Supplementary Table S1. Survival rates of the mice.

Supplementary Figure S1. Electrophoretic profiling of the effect of the purified SrtA protein via SDS–PAGE.

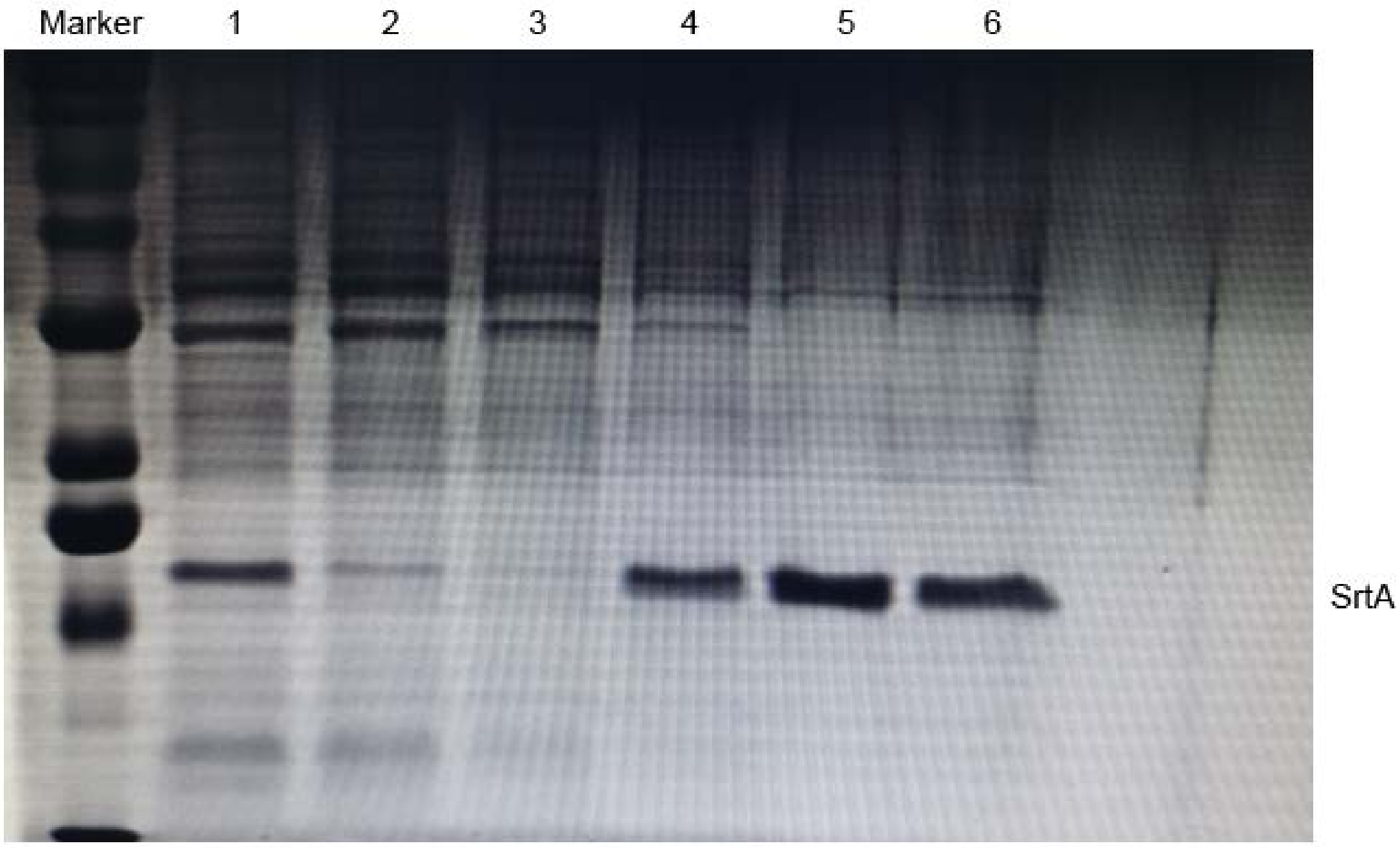

Supplementary Figure S1. Electrophoretic profiling of the effect of the purified SrtA protein via SDS–PAGE. Lanes 1 and 2 show the BL21 (DE3) pET28a::SrtA protein expression postinduction and postinduction with IPTG, respectively. Lane 3 highlights nonspecific proteins eluted at a low imidazole concentration (10 mM). Proteins collected at a moderate imidazole concentration (50 mM) are shown in lane 4, while lanes 5 and 6 display the target SrtA protein purified under stringent conditions using 100 and 200 mM imidazole, respectively.

Supplementary Figure S2. The impact of different concentrations of verbascoside on RBC integrity.

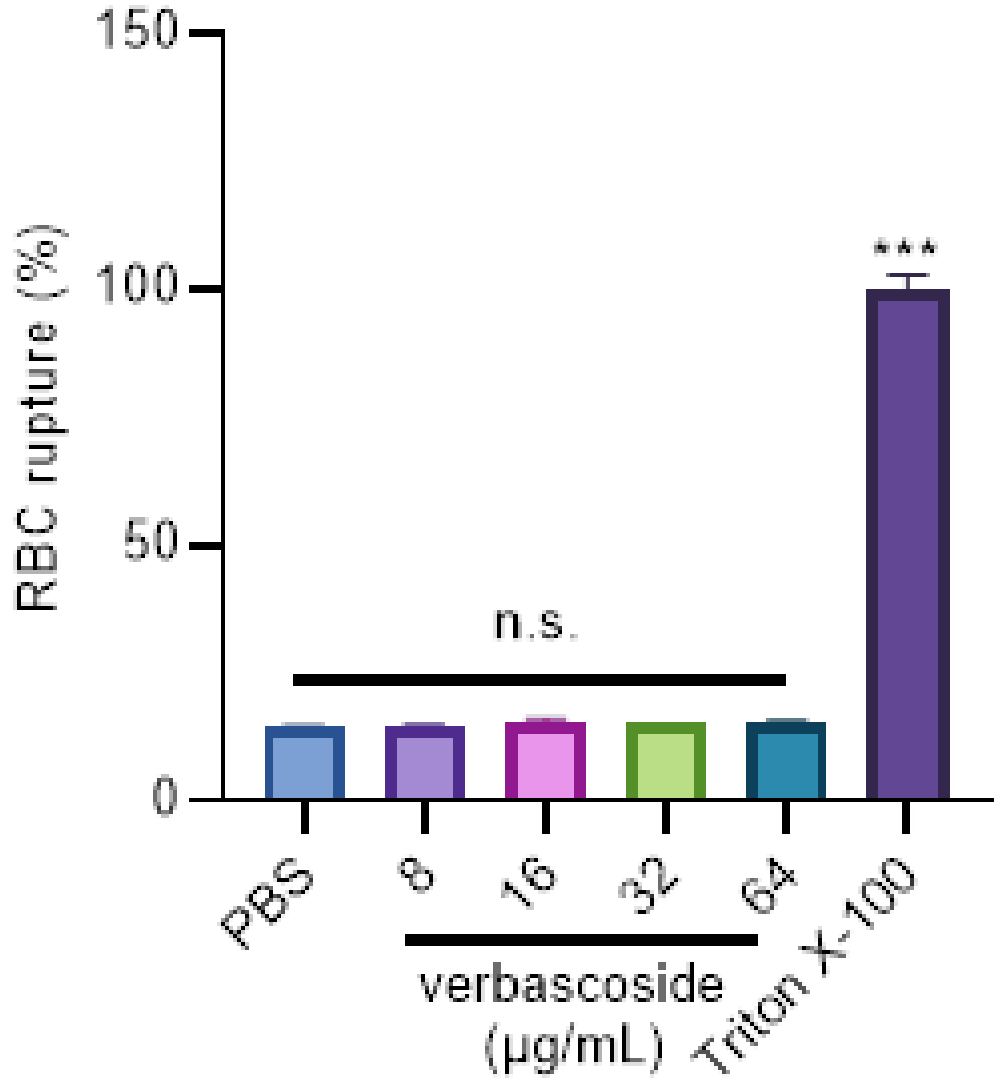

Supplementary Figure S3. The MIC of verbascoside against standard and clinical strains of *S. aureus* stained with resazurin.

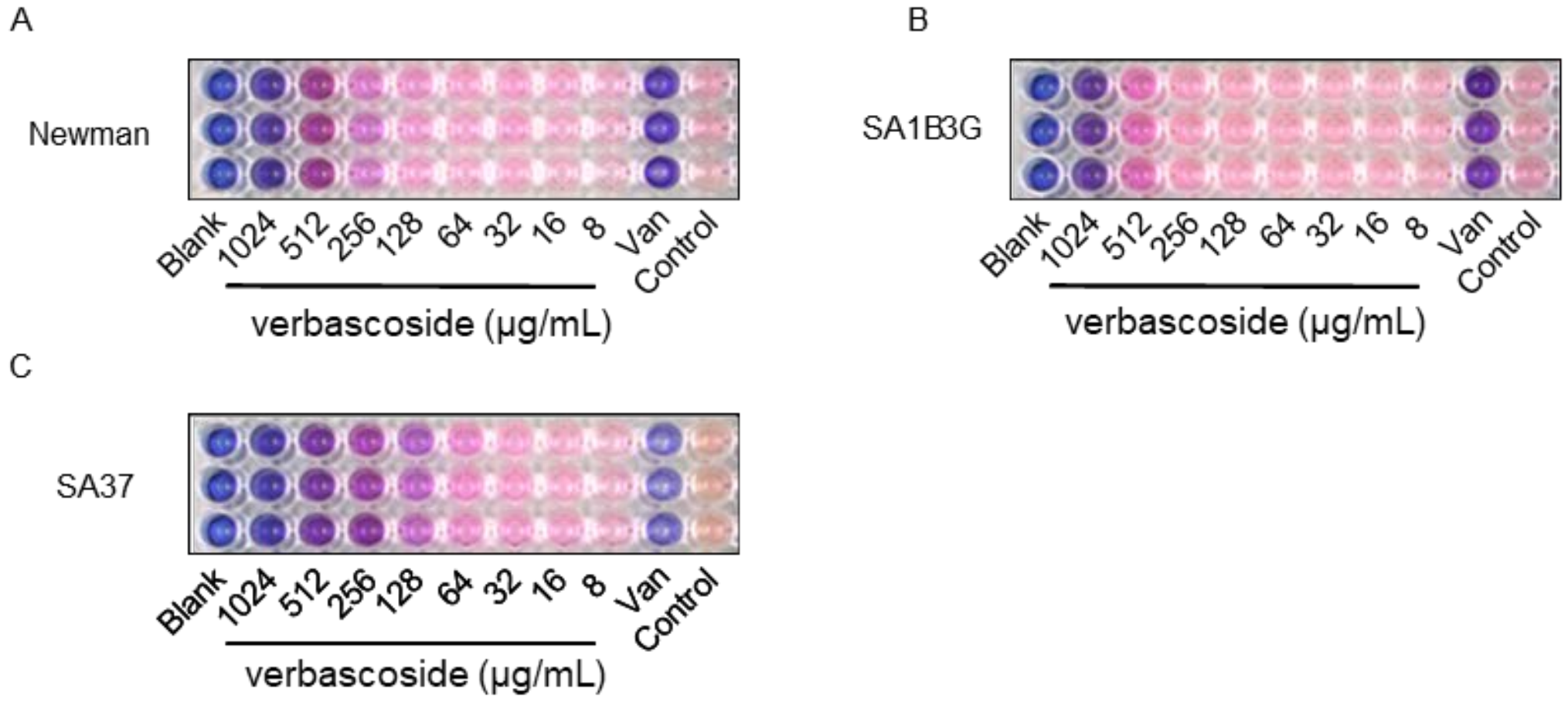

Fig S1. The MIC of verbascoside against standard and clinical strains of *S. aureus* stained with Resazurin. (A) The MIC of verbascoside against the standard strain of *S. aureus* Newman. (B) The MICs of verbascoside against clinical strains of *S. aureus*, including the SA1B3G strain (C) and SA37.

Supplementary Figure S4. Quantitative assessment of *S. aureus* biofilm formation under verbascoside exposure using crystal violet staining.

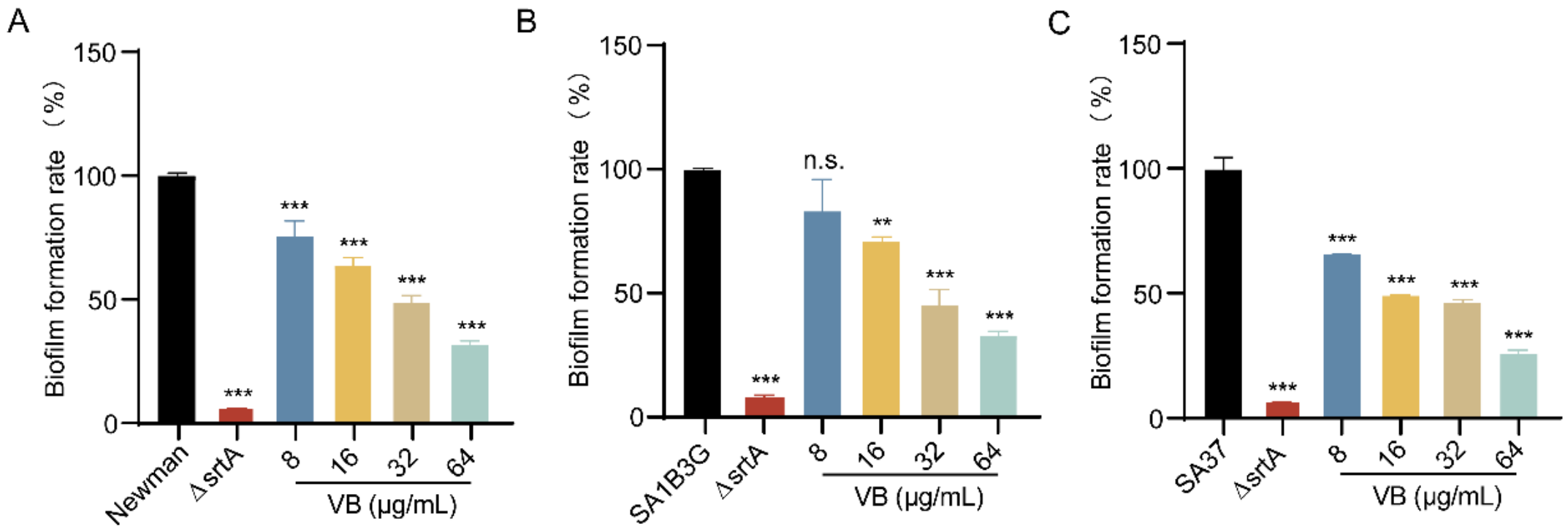

Figure S4. Quantitative assessment of *S. aureus* biofilm formation under verbascoside exposure using crystal violet staining. (A) Impact of verbascoside on biofilm formation by *S. aureus* Newman, (B) SA1B3G, and (C) SA37.

Supplementary Figure S5. Impact of verbascoside on the survival rate of *S. aureus* USA300 Δ*srtA* infected with *Galleria mellonella*.

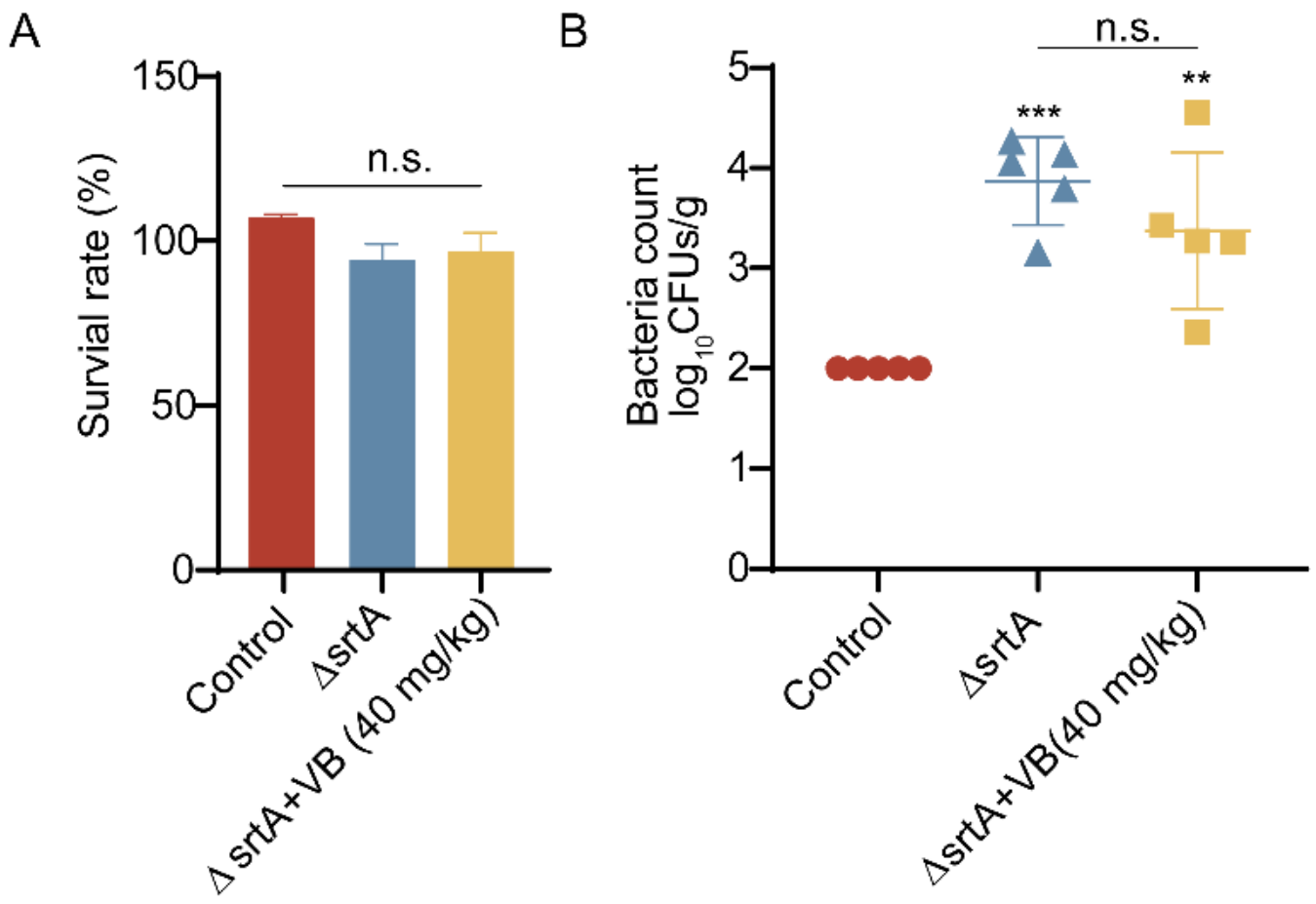

Figure S5. Impact of verbascoside on the survival rate of *S. aureus* USA300 Δ*srtA* infected with *Galleria mellonella*. (A) A comparative investigation revealed distinct cohorts of *Galleria mellonella* (n = 10), comprising an untreated control group (Δ*srtA*) and a treated group (Δ*srtA*+VB) administered verbascoside (40 mg/kg). The survival rates of these cohorts were documented at 120 hours postinfection. (B) Enumeration of colony-forming units (CFUs) in *Galleria mellonella* larvae following infection (n = 5), determined using the agar dilution method.

Supplementary Figure S6. Treatment of mice with verbascoside-induced *S. aureus* USA300 Δ*srtA* strain-infected pneumonia.

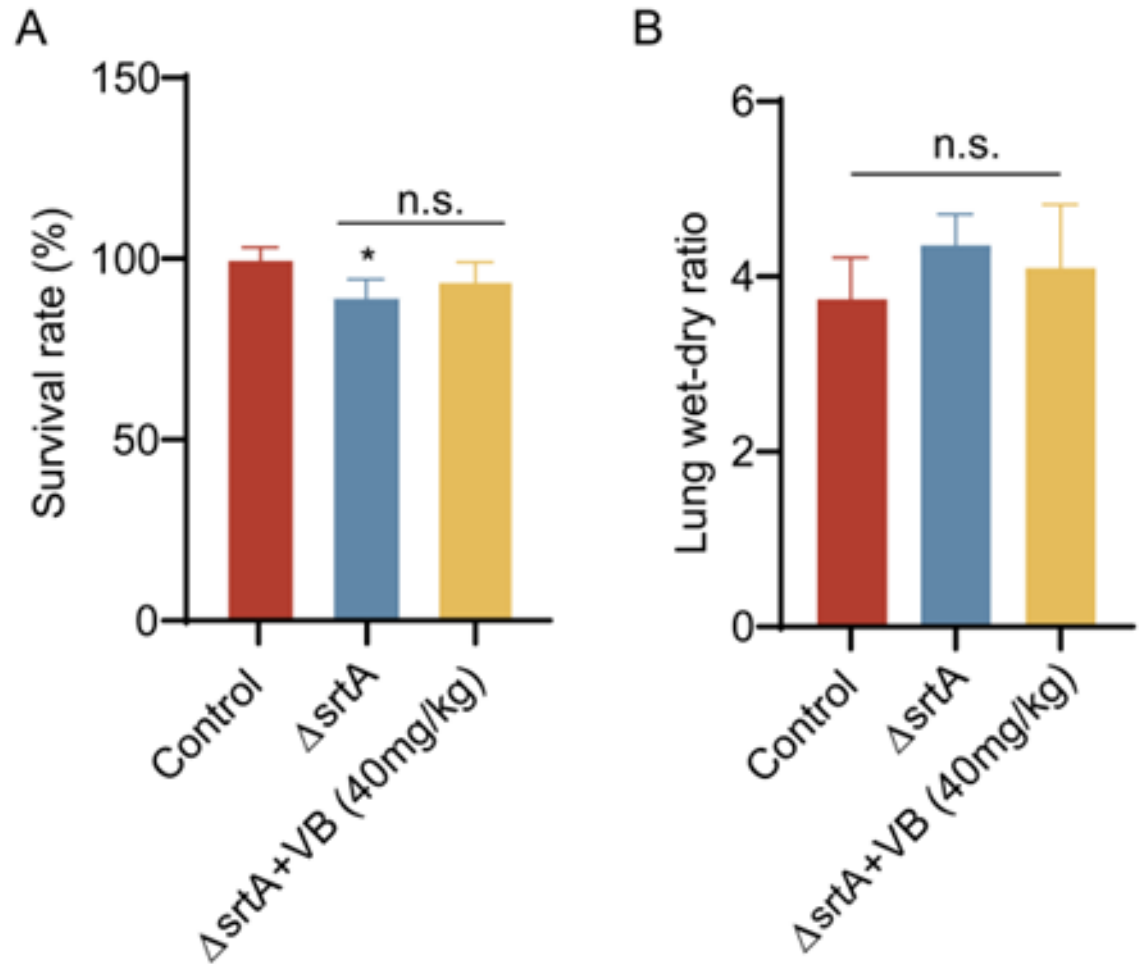

Figure S6. Treatment of mice with verbascoside-induced *S. aureus* USA300 Δ*srtA*-infected pneumonia. (A) Assessment of the impact of verbascoside on the survival rate of mice (n = 10) exposed to a lethal dose of the *S. aureus* USA300 Δ*srtA*. There was no significant difference in survival between the verbascoside-treated Δ*srtA* group and the untreated Δ*srtA* group. (B) Evaluation of the lung dry-to-wet weight ratio as an indicator of the therapeutic efficacy of verbascoside in *S. aureus* USA300 Δ*srtA*-induced pneumonia in mice (n=6).

Supplementary Table S1. Survival rates of the mice.

Mice survival (%)							
Groups	Day 1	Day2	Day 3	Day 4	Day 5	Day 6	Day 7
Noninjected	100	100	100	100	100	100	100
Control (PBS)	100	100	100	100	100	100	100
Verbascoside 20 mg/kg	100	100	100	100	100	100	100
Verbascoside 40 mg/kg	100	100	100	100	100	100	100
